# Editorial: Vegetarian Dietary Patterns in the Prevention and Treatment of Disease

**DOI:** 10.3389/fnut.2020.00092

**Published:** 2020-06-19

**Authors:** Hana Kahleova, David L. Katz

**Affiliations:** ^1^Physicians Committee for Responsible Medicine, Washington, DC, United States; ^2^True Health Initiative, Derby, CT, United States; ^3^Prevention Research Center, Yale University/Griffin Hospital, Derby, CT, United States

**Keywords:** vegetarian, plant-based, dietary pattern, prevention, treatment

Dietary patterns that emphasize the consumption of vegetables, fruits, whole grains and legumes, are recognized for their health-promoting properties (1). Such diets encompass vegetarian and vegan types, while extending to diverse, plant-predominant dietary patterns (2). Such diets are typically rich in fiber, antioxidants and phytochemicals, but much lower in saturated fat, added sugar, animal protein, and sodium compared to more conventional dietary patterns (3). The current 2015-2020 *Dietary Guidelines for Americans* recommend a vegetarian dietary pattern as one of three healthy dietary patterns, along with the Mediterranean and healthy U.S. style dietary patterns (4). Minimally processed plant foods are emphasized in all three, while only the vegetarian diet excludes meat entirely.

When examining factors that do drive overall, objective measures of diet quality, there are two salient influences: shifts in animal vs. plant food, and shifts from highly processed to unprocessed/minimally processed foods (5). Other things being equal, in modern countries where overt protein or calorie malnutrition is a rare concern, shifts to plant foods correlate consistently and robustly with higher overall diet quality. Other things being equal in nearly any dietary context, shifts from highly processed to less processed foods do the same. Discussions here presuppose the above influences and pertain to high-quality vegetarian diets.

Cardiovascular disease is the leading global cause of mortality, being responsible for 46% of non-communicable disease deaths (6). It has been estimated that about 85.6 million Americans are living with some form of CVD, the prevalence of which continues to rise (7). This increase has been linked to lifestyle factors, particularly low overall diet quality and lack of exercise (8). The systematic review and meta-analysis of prospective cohort studies by Glenn et al. showed that self-reported vegetarian dietary pattern was associated with a 22% lower cardiovascular mortality and a 28% lower incidence of cardiovascular disease, which is an effect comparable to a combination of the most current pharmacotherapies (9). This implies that in the study population, vegetarianism involves not only the avoidance of meat, but a general elevation of diet quality. The benefits associated with high-quality vegetarian diets come without the unwanted side effects and for a much lower market price than standard pharmacotherapy aimed at comparable risk reduction. Furthermore, plant-based eating may also help reduce society's health care costs, such as hospital admissions and doctor's bills, as well as increasing the number of healthy years filled with productivity. Thus, plant-based diets could save billions of dollars in health care costs (7, 10).

It is also important to note that environmental and climate effects of food at scale are relevant to health outcomes- both directly and indirectly. Here, too, there are important implications of vegetarianism. Shifts from animal to plant-food-predominant diets are key recommendations at the interface of human and planetary health (11).

Allen et al. described a case study of a 54-year old woman who not only reversed her diabetes, but also her systolic dysfunction, increasing her ejection fraction from 21 to 55%, after following a plant-based diet for <6 months. Plant-based diets improve the risk factors for heart failure, but also have direct benefits on heart metabolism and function (12, 13). Given the severe prognosis of heart failure, even limited evidence for benefits from plant-based diets warrants careful attention.

What role does fruit play in weight management? Guyenet performed a systematic review of randomized controlled studies and demonstrated that whole, fresh fruit promotes weight maintenance or modest weight loss and may be therefore used as a part of a healthy diet in the prevention and treatment of excess body weight and adiposity. These findings support the current recommendations by the U.S. Department of Agriculture (4) to increase fruit consumption, which may have a positive impact on public health and obesity control.

Plant-based diets have been shown to be beneficial in a number of different populations. Davis et al. have conducted a randomized controlled trial to test the effectiveness of a plant-based diet and moderate exercise in people with type 2 diabetes in the Republic of the Marshall Islands. The study is still ongoing and it will be exciting to see if diabetes can be significantly improved or even reversed in this part of the world. Singh et al. have shown that plant-based eating is associated with BMI within the recommended range in Hispanic/Latino Seventh-day Adventists. This finding has major implications because currently, diabetes rates are 60% higher among Hispanics/Latinos compared with non-Hispanic Whites (14). Finally, our children and adolescents are the future of our nation. What is the quality of their diet? Would a vegetarian diet promote their health as much as in adults? Are teenagers able to maintain a healthy vegetarian diet? Segovia-Siapco et al. demonstrated that vegetarian adolescents had a more favorable dietary intake profile than non-vegetarians, eating more vegetables, fruits, nuts, legumes and soy products, and much less saturated fat. Such dietary habits likely translate into long-term risk reduction, and prevention of chronic disease.

Alwarith et al. pulled together a review paper on the potential use of plant-based diets for rheumatoid arthritis. This chronic autoimmune disease affects about 1% of the world's population and leads to inflammation, pain, and eventually permanent joint damage (15). The paper presents encouraging evidence that plant-based diets may play an important role in the management and/or remission of rheumatoid arthritis, although some additional trigger foods might need to be eliminated from the diet. The improvements in response to a plant-based diet may be at least partly explained by the changes in gut microbiome.

Tomova et al. described the benefits of vegetarian and vegan diets on gut microbiota. Plant-based diets seem to promote a more diverse and stable gut ecosystem and increase the short-chain fatty acid (SCFA) producing bacteria. The SCFAs improve immune function, blood-brain barrier integrity, increase insulin sensitivity and have cardioprotective effects.

The health benefits of plant-predominant diets have been observed throughout the whole spectrum, from strict vegan diets all the way to much more liberal lacto-ovo-vegetarian and even semi-vegetarian diets. Due to the design of pertinent studies, it is not always possible to distinguish and quantify the health benefits of any of these dietary patterns relative to another at comparable levels of overall quality.

The range of plant-predominant dietary patterns may be recommended for health promotion without population-specific precautions appended. According to the Academy of Nutrition and Dietetics, vegetarian, and vegan diets are appropriate for every stage of life including pregnancy, infancy, and childhood, as well as for athletes. Reliable sources of vitamin B-12 need to be secured (16); these can be food sources, or supplements.

Pesticides and herbicides are still widely used in food plant cultivation. Most pesticides are lipophilic and therefore accumulate in fat, particularly in animals feeding upon these foods for prolonged periods of time (17). Thus, the risks of exposure to these compounds at harmful levels is greater with animal than with plant food consumption. The bioaccumulation of pesticides can have harmful effects on animals, as well as people who consume meat and dairy. Organic foods contain lower levels of pesticides, and also higher levels of certain nutrients (18), but may not be affordable or available for everyone.

Regarding costs, fresh produce is often expensive relative to other available food choices. However, some staple foods in plant-predominant diets, such as rice and other cooking grains, beans, cabbage, and potatoes, are much less expensive than meat, dairy, and most processed foods. Water, of course, is either freely available or inexpensive relative to sugar-sweetened beverages. Beans and lentils are highly nutritious substitutes for animal-food protein; are considerably less expensive; and offer environmental impact benefits as well. Thus, there are many opportunities to adopt a more plant-predominant dietary pattern, and improve overall diet quality, unimpeded by barriers of cost.

Arguments for diverse health benefits of high-quality vegetarian diets are thus varied and robust. They are, as well, timely- as meat consumption is a major consideration in an array of threats to planetary health (19, 20). Loss of rainforest in the Amazon, for instance, is directly attributable to the global appetite for meat. The assembly of papers collected here highlights direct health benefits of well-practiced vegetarianism at a time, and in a context, when the indirect benefits- via effects on aquifers, ecosystems, fires, floods, and droughts- may be even more salient.

## Author Contributions

All authors listed have made a substantial, direct and intellectual contribution to the work, and approved it for publication.

## Conflict of Interest

The authors declare that the research was conducted in the absence of any commercial or financial relationships that could be construed as a potential conflict of interest.
